# Exogenous Indole-3-Acetic Acid Induced Ethanol Tolerance in Phylogenetically Diverse Saccharomycetales Yeasts

**DOI:** 10.1264/jsme2.ME21053

**Published:** 2022-01-27

**Authors:** Ruo-Ting Hsiung, Ming-Chung Chiu, Jui-Yu Chou

**Affiliations:** 1 Department of Biology, National Changhua University of Education, Changhua 500, Taiwan

**Keywords:** indole-3-acetic acid (IAA), exogenous growth regulatory signal, ethanol tolerance, *Saccharomyces cerevisiae*, make-accumulate-consume strategy

## Abstract

Indole-3-acetic acid (IAA) is an exogenous growth regulatory signal that is produced by plants and various microorganisms. Microorganisms have been suggested to cross-communicate with each other through IAA-mediated signaling mechanisms. The IAA-induced tolerance response has been reported in several microorganisms, but has not yet been described in Saccharomycetales yeasts. In the present study, three common stressors (heat, osmotic pressure, and ethanol) were examined in relation to the influence of a pretreatment with IAA on stress tolerance in 12 different lineages of *Saccharomyces cerevisiae*. The pretreatment with IAA had a significant effect on the induction of ethanol tolerance by reducing the doubling time of *S. cerevisiae* growth without the pretreatment. However, the pretreatment did not significantly affect the induction of thermo- or osmotolerance. The IAA pretreatment decreased the lethal effects of ethanol on *S. cerevisiae* cells. Although yeasts produce ethanol to outcompete sympatric microorganisms, IAA is not a byproduct of this process. Nevertheless, the accumulation of IAA indicates an increasing number of microorganisms, and, thus, greater competition for resources. Since the “wine trait” is shared by both phylogenetically related and distinct lineages in Saccharomycetales, we conclude that IAA-induced ethanol tolerance is not specific to *S. cerevisiae*; it may be widely detected in both pre-whole genome duplication (WGD) and post-WGD yeasts belonging to several genera of Saccharomycetales.

Indole-3-acetic acid (IAA) is an auxin that regulates plant physiology and growth (Spaepen *et al.*, 2007; Liu *et al.*, 2016). Several organisms, including plants, bacteria, algae, yeast, and multicellular fungi, produce and respond to IAA in the environment (Spaepen *et al.*, 2007; Reineke *et al.*, 2008; Fu *et al.*, 2015). Previous studies demonstrated that microbial IAA increases seed germination and root elongation (Geldner *et al.*, 2003; Teale *et al.*, 2006; Contreras-Cornejo *et al.*, 2009; Spaepen and Vanderleyden, 2011), whereas plant IAA regulates physiological responses by plant-associated microorganisms, such as inhibiting growth and *vir* gene expression (Spaepen *et al.*, 2007). These interspecific effects make IAA a diffusible signal that elicits communication among different organisms (Fu *et al.*, 2015).

An increased environmental IAA concentration indicates the aggregation of microorganisms and greater competition (Geldner *et al.*, 2003; Prusty *et al.*, 2004; Bianco *et al.*, 2006; Teale *et al.*, 2006; Reineke *et al.*, 2008; Contreras-Cornejo *et al.*, 2009; Imperlini *et al.*, 2009; Spaepen and Vanderleyden, 2011; Donati *et al.*, 2013; Kulkarni *et al.*, 2013; Sun *et al.*, 2014; Fu *et al.*, 2015; Liu *et al.*, 2016; Chung *et al.*, 2018; Lin *et al.*, 2020). Under favorable growth conditions, the addition of exogenous IAA was shown to reduce the growth rate of *S. cerevisiae* (Prusty *et al.*, 2004; Liu *et al.*, 2016), but also increased it in the presence of relatively low concentrations of *Aureobasidium pullulans* (625‍ ‍μM), *Cryptococcus flavus* (312.5‍ ‍μM), and *Fusarium delphinoides* (<50‍ ‍μM) (Kulkarni *et al.*, 2013; Sun *et al.*, 2014). We previously demonstrated that relatively high IAA concentrations inhibited the growth of the green algae *Desmodesmus* spp. and promoted the accumulation of lipid droplets in algal cells, an effect that is related to stress tolerance (Chung *et al.*, 2018). Follow-up studies confirmed that stress tolerance was enhanced in green algae treated with IAA (Lin *et al.*, 2020). Similar mechanisms have been detected in bacteria. Increases were observed in trehalose, lipopolysaccharide, exopolysaccharide (EPS), and biofilm production in *Escherichia coli* treated with IAA (Bianco *et al.*, 2006). Furthermore, IAA triggered enhanced tolerance to stress and toxic compounds, which correlated with the up-regulated expression of the heat shock protein DnaK. Similarly, a treatment with IAA stimulated biofilm formation and EPS production in *Bradyrhizobium japonicum*, a nitrogen-fixing endosymbiont of soybean (Donati *et al.*, 2013). Imperlini *et al.* (2009) demonstrated that IAA increased the survival and symbiotic nitrogen fixation ability of *Sinorhizobium meliloti* (Imperlini *et al.*, 2009). Collectively, these findings suggest that IAA acts as a phytohormone and also regulates metabolic levels in microorganisms. However, even though IAA is a common growth regulator in fungi, its effects on stress tolerance have not yet been characterized in detail.

The budding yeast *Saccharomyces cerevisiae* is a single-celled fungus that is commonly used as a model organism in eukaryotic studies (Duina *et al.*, 2014). It is widely distributed and may be isolated from diversified sources (Liti *et al.*, 2009; Duan *et al.*, 2018; Peter *et al.*, 2018). One of the most prominent features of *S. cerevisiae* is its ability to rapidly convert sugars to alcohol. This wine trait has been hypothetically traced to the end of the Cretaceous period, concomitant with the origin of angiosperms, and helps the budding yeast outcompete its microbial competitors (mainly bacteria) in high-sugar environments (Dashko *et al.*, 2014). However, ethanol negatively affects Saccharomycetales yeast by decreasing its growth rate and cell viability. Ethanol tolerance in different Saccharomycetales yeasts ranges between 6 and 14%, as reported by Rozpędowska *et al.* (2011). Osmotic stress associated with alcohol secretion also dehydrates yeast cells. During fermentation, marked changes in environmental conditions cause fluctuations in the amount of stress (Birch and Walker, 2000; Stanley *et al.*, 2010). Temperature fluctuations are also very common in natural environments. Therefore, thermal adaptation is essential for the maintenance of thermal homeostasis and is also required for virulence in yeasts and other opportunistic pathogenic fungi (Nicholls *et al.*, 2011; Brown *et al.*, 2014). Microbes have evolved protective mechanisms against harmful stresses induced by diverse environmental conditions.

In the present study, we investigated whether a pretreatment with IAA promoted enhanced stress-tolerant phenotypes in *S. cerevisiae*. We also examined IAA-induced ethanol tolerance in both phylogenetically related and distinct Saccharomycetales yeasts. Several modern Saccharomycetales yeasts, apart from *S. cerevisiae* and its sister species, often occupy a similar niche and have equivalent capacities to produce and accumulate ethanol (Merico *et al.*, 2007). These diverse yeast lineages have been suggested to contain independently used global promoter rewiring as part of the “make-accumulate-consume” strategy (Rozpędowska *et al.*, 2011). The premise of this strategy is that Saccharomycetales yeasts ferment glucose to defend sugar-rich resources (such as fruit) from competitors by exploiting the toxicity of ethanol. These yeasts are subsequently able to consume ethanol after establishing competitive dominance within their ecological niche (Hagman *et al.*, 2013). This particular life strategy has been suggested to have evolved as a multi-step process that predates the whole genome duplication (WGD) event that hypothetically occurred ∼100 million years ago, possibly near the origin of all flowering plants (Piškur *et al.*, 2006). Therefore, we also examined several pre-WGD and post-WGD Saccharomycetales yeasts, which may occupy similar niches in nature and rely on glucose as their primary substrate, and analyzed their ethanol tolerance and cell viability under IAA-induced ethanol tolerance.

## Materials and Methods

### Yeast source and culture conditions

To achieve the greatest coverage of the phylogenetically diverged lineages of *S. cerevisiae*, 12 different lineages were selected for the present study by refereeing to the phylogenetic tree reconstructed by Liti *et al.* (2009). Other than *S. cerevisiae*, *S. paradoxus*, *S. eubayanus*, *Kazachstania servazzii*, *Zygosaccharomyces bisporus*, and *Z. rouxii* belonging to the post-WGD group and *Torulaspora* sp., *Kluyveromyces marxianus*, *Kluyveromyces* sp., *Dekkera bruxellensis* belonging to the pre-WGD group were used in the present study. These Saccharomycetales yeasts were provided by different resources. *S. cerevisiae* strains were provided by Dr. Gianni Liti (Université Côte d’Azur, CNRS, INSERM, IRCAN, Nice, France) and Dr. Jun-Yi Leu (Institute of Molecular Biology, Academia Sinica, Taiwan); *S. paradoxus* and *S. eubayanus*, which were examined in Greig *et al.* (2002), were provided by Dr. Duccio Cavalieri (Fondazione Edmund Mach, San Michele all’Adige, Italy). The other yeasts were isolated by the authors of the present study, including *Ka. servazzii* and *Kl. marxianus* from milk kefir (Hsu and Chou, 2021), *Z. bisporus* from coffee cherry (unpublished data), *Z. rouxii* from fermented vinegar (Hsu and Chou, 2021), *Torulaspora* sp. from living *Drosera spatulata* (Fu *et al.*, 2016), *Kluyveromyces* sp. from the rhizosphere of cherry tomato (unpublished data), and *Dekkera bruxellensis* from a kombucha culture (unpublished data) (Supplementary Table 1). The yeasts isolated by the authors of this study were identified by sequencing their rDNA regions (SSU-ITS-LSU) and isolates are available upon request from the corresponding author. All yeast isolates were maintained in yeast extract–peptone–dextrose (YPD; 1% yeast extract, 2% peptone, and 2% dextrose) medium and incubated in the dark on a shaker at 28°C and 150 revolutions min^–1^.

### Stress tolerance test

The effects of IAA on osmo-, thermo-, and ethanol tolerance were examined. *S. cerevisiae* was cultured at 28°C overnight in YPD medium and refreshed for 3 h. IAA is hypothesized to be a signal for the accumulation of competitive microbes. In competition for sugar resources, yeasts produce ethanol to suppress the growth of sympatric microbes. Since the accumulation of sympatric microbes is accomplished by increases in IAA concentrations, IAA may stimulate yeast to trigger tolerance to unpredicted fluctuations. Therefore, to focus on the early stimulation of tolerance, we pretreated *S. cerevisiae* with IAA before its growth under stress conditions. Regarding the IAA pretreatment, 20‍ ‍μL of a log-phase yeast suspension was added to 3‍ ‍mL YPD medium with a serial dose of IAA (0, 300, 600, 900, or 1,200‍ ‍μM) and incubated at 28°C for 24 h. After the pretreatment, yeast suspensions were adjusted to a density of ~1.28×10^6^‍ ‍cells‍ ‍mL^–1^ in YPD medium by a 10-fold dilution of the suspensions with the corresponding optical density (0.81) of 660‍ ‍nm. To remove IAA from the medium, 3‍ ‍mL of the yeast suspension was centrifuged at 13,000×*g* for 1‍ ‍min, the supernatant was discarded, the pellet was washed with 3‍ ‍mL sterile water, centrifuged again at 13,000×*g* for 1‍ ‍min, the supernatant was discarded, and the yeast pellet was finally suspended in 200‍ ‍μL YPD medium.

To test osmo- and ethanol tolerance, prepared yeast suspensions were examined in 3‍ ‍mL YPD medium at 28°C with 1 M NaCl and 11% (v/v) ethanol, respectively. To assess thermo-tolerance, prepared yeast suspensions were examined in 3‍ ‍mL YPD medium at 40°C because the majority of Saccharomycetales yeast species grow optimally below 35–37°C and die at high temperatures (>50°C). All examinations were conducted using 3 sets of yeast suspensions and were repeated twice.

Yeast growth was monitored at 0, 12, 24, 36, and 48 h by measuring absorbance at 660‍ ‍nm with a microplate reader (Thermo Scientific Multiskan GO). The doubling time (*T_d_*, min), calculated with the equation *T_d_*=*t*/log_2_(*f_2_*/*f_1_*), was used to represent growth efficiency. In this equation, *t* represents the reaction time (the length of time yeast cell growth remained in the log phase: 12 h for no stress, 24 h for heat and osmotic stress, and 36 h for ethanol stress); *f_1_* represents the initial number of cells; and *f_2_* represents the final number of cells.

### Quantitative assessment of the yeast cell death rate during ethanol stress

After 50% of the *S. cerevisiae* strains tested were found to be more tolerant to ethanol stress when pretreated with IAA, we examined the effects of a pretreatment with IAA (900‍ ‍μM) on the cell death rate. Cell death was detected using the apoptosis fluorescent dye SYTOX Green (Invitrogen^TM^, Life Technologies, S7020). Yeast cells were cultured in YPD medium with 11% (v/v) ethanol and prepared as described above. At 0, 12, and 15 h, 1‍ ‍mL of each yeast culture was transferred to a 1.5-mL microtube and centrifuged at 13,000–16,000×*g* for 1‍ ‍min. The supernatant was then discarded and the sample was washed with phosphate-buffered saline (PBS). Samples were vortexed with 10‍ ‍μL of PBS and 1‍ ‍μL SYTOX Green in the dark for 10‍ ‍min. Samples were observed and photographed using a Leica DM2500 fluorescence microscope, and 200–500 cells from each sample were randomly counted to assess the death rate.

### Effects of IAA on ethanol tolerance by a cell viability assay

We performed a cell viability assay using two post-WGD and four pre-WGD genera of Saccharomycetales to establish whether the inhibition of lethal effects following IAA-induced ethanol tolerance is common in Saccharomycetales yeasts (Supplementary Table S1). The 24-h yeast inocula were refreshed with YPD medium for 3 h, and 10‍ ‍μL of log-phase yeast cells was then transferred to YPD medium with or without 900‍ ‍μM IAA for 2 days. After the incubation, approximately 3×10^6^ cells were transferred into 3‍ ‍mL YPD medium with 11 or 13% (v/v) ethanol and incubated until analyzed. Five microliters of the yeast suspension was spotted onto YPD agar plates and observed after 0, 4, 8, 12, and 24 h. Images were taken once distinct colonies of the samples that were spotted at the last time point were observed (approximately 24–48 h after the inoculation). The abundance of the colonies was monitored at each of the time points to compare the effects of the IAA pretreatment on the growth of Saccharomycetales yeasts.

### Statistical ana­lysis

We modeled the effects of IAA titers on the doubling time of each *S. cerevisiae* strain pretreated with an environmental stress (or without stress) using a general linear mixed model (LMM). We considered doubling time to be a response variable, IAA titer to be a fixed factor variable, and the two experimental replications to be random factors. The significance of the fixed factor variable was assessed by an ana­lysis of variance (ANOVA). In further examinations to clarify whether changes to doubling time were induced by stress or by a general response to IAA, we added an interaction term to the two-factor LMM to investigate the parallel response of yeast pretreated with and without stress to IAA. This model is similar to the previous one, with stress as an additional fixed factor variable, and an interaction term in the two fixed factor variables. We tested the significance of the interaction term in the two fixed factor variables using ANOVA. To understand the general response of the IAA pretreatment on the 12 *S. cerevisiae* strains under each type of stress (osmotic, thermal, and ethanol), we calculated tolerance levels using the observed doubling time ratio in the following formula: (*D_t_*–*D_c_*)/(*D_t_*+*D_c_*). In this formula, *D_t_* represents the doubling time of IAA-pretreated yeast, and *D_c_* represents the mean doubling time of non-IAA-pretreated yeast. doubling time ratio values fell in the range of –1 to 1, with a ratio of 0 indicating that the IAA pretreatment had no effects on stress tolerance. The significance of the doubling time ratio compared to 0 was calculated using the Mann–Whitney *U* test. The cell death rate was analyzed by Fisher’s exact test to assess the significance of the IAA pretreatment.

Model-building and hypothesis tests were conducted using basic functions and the “ImerTest” package in R (R Core Team, 2019).

## Results

### Stress tolerance assay

**Fig. 1.**
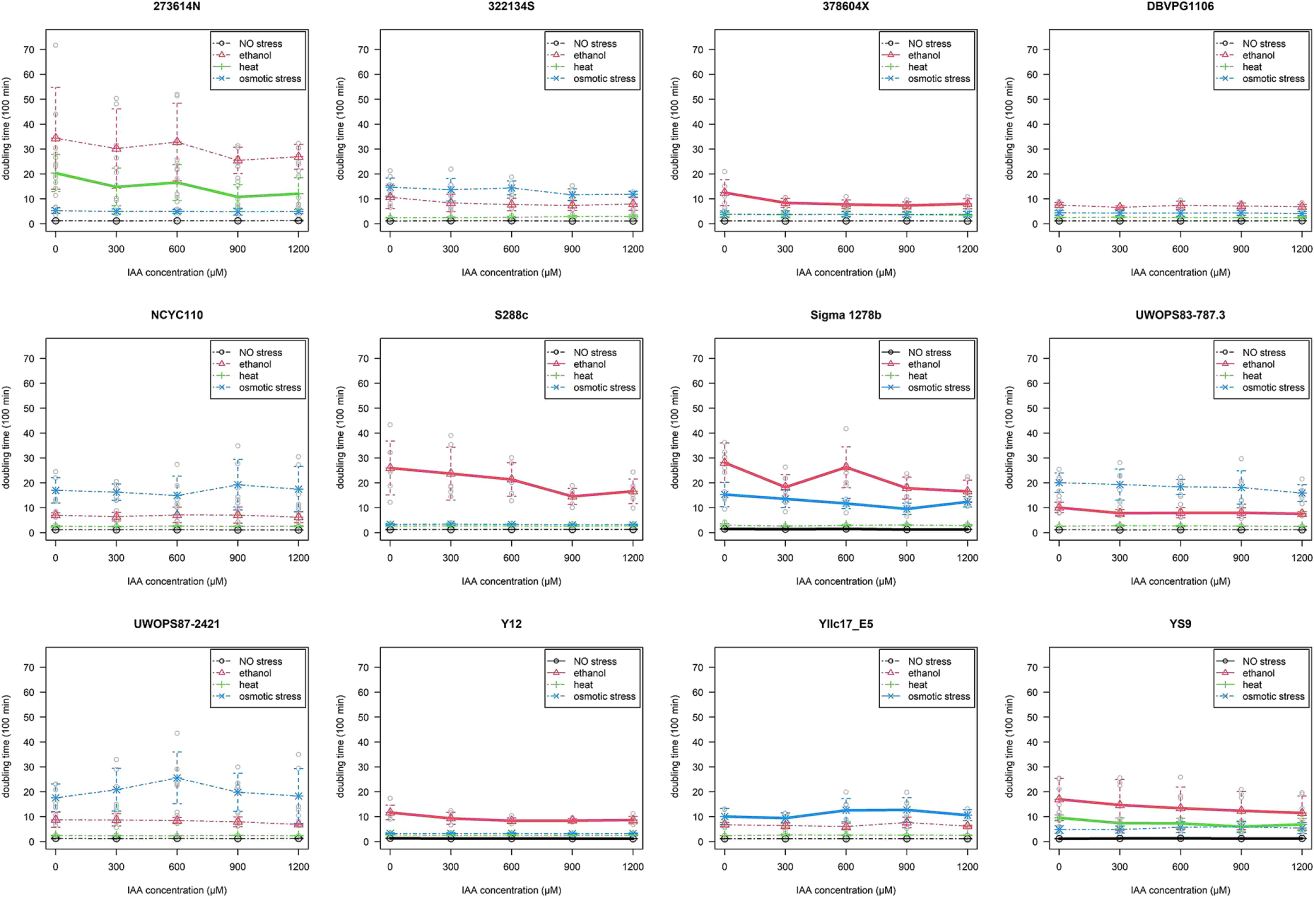
Doubling time of *Saccharomyces cerevisiae* strains pretreated with a series of indole-3-acetic acid (IAA) titers under three environmental stresses (ethanol, heat, and osmotic). Solid lines indicate the significant effects of the IAA pretreatment on the doubling time, as assessed by ANOVA.

All *S. cerevisiae* strains cultured with environmental stress grew more slowly, as shown by longer doubling times, than those cultured without stress. However, stress tolerance significantly varied among strains according to the IAA pretreatment (Fig. 1). Among the 12 *S. cerevisiae* strains pretreated with IAA, some showed a decreased doubling times under specific conditions, including the two yeast strains (Sigma 1278b and Y12) that were not exposed to any environmental stress, six strains (378604X, S288c, Sigma 1278b, UWOPS83-787.3, Y12, and YS9) that were treated with ethanol, two strains (273614N and YS9) that were treated with heat, and one strain (Sigma 1278b) that was treated with osmotic stress. Only two strains showed an increased doubling time in the IAA pretreatment: YS9, which was not exposed to environmental stress, and Yllc17_E5, which was treated with osmotic stress (Fig. 1 and Table 1). Despite some strains being less responsive to the highest (1200‍ ‍μM) IAA titer (*e.g.*, Yllc17_E5 with osmotic stress), most strains displayed a significantly shorter doubling time at the second highest (900‍ ‍μM) IAA titer (Fig. 1). In some strains, the significance of the IAA pretreatment was disputed because of the lack of significance of the interaction term (*e.g.*, YS9 with ethanol stress, 273614N with heat stress, and Yllc17_E5 with osmotic stress), which implied that the effects of IAA on doubling time were not attributed to stress tolerance, but were rather a general effect.

At all examined titers, the pretreatment with IAA significantly induced ethanol tolerance, but did not significantly affect osmo- or thermotolerance (Fig. S1). Among the six strains displaying IAA-induced ethanol tolerance, the maximum reduction in doubling time was observed at the higher titers (600–1,200‍ ‍μM) of the IAA pretreatment at which ratios ([*Dt*–*Dc*]/[*Dt*+*Dc*]) ranged between –0.282 (in S228c) and –0.143 (in UWOPS83-787.3).

### IAA pre-exposure reduced ethanol-related cell death

**Fig. 2.**
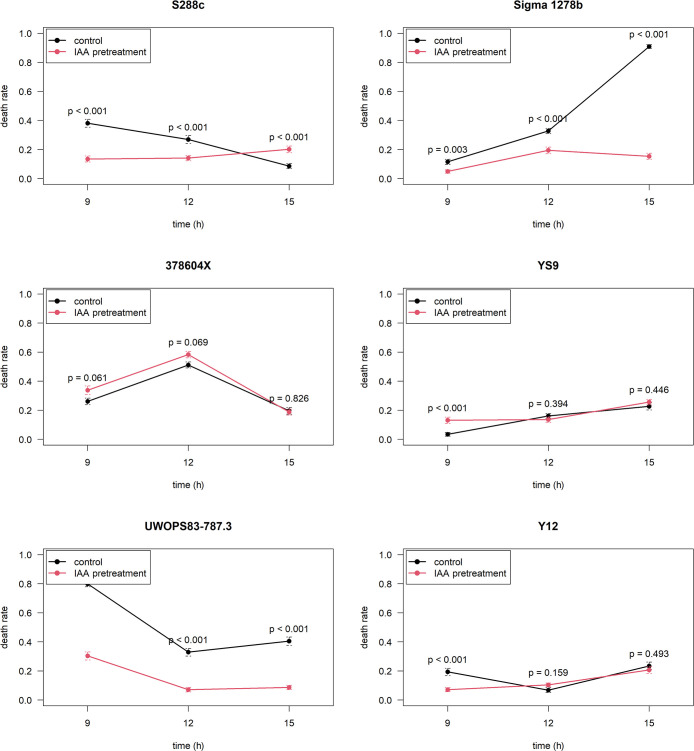
Cell death rate of *Saccharomyces cerevisiae* strains pretreated with indole-3-acetic acid (IAA; red line) or the control (black line) and cultured in ethanol as an environmental stress. *P* values indicate significance as assessed by Fisher’s exact test.

The pretreatment of yeast cultures with IAA induced ethanol tolerance in six *S. cerevisiae* strains, suggesting that the IAA pretreatment stimulated an adaptive response by alleviating cell death. Among the six strains, four (S288c, Sigma 1278b, UWOPS83-787.3, and Y12) had a significantly lower cell death rate on at least one time point after the IAA pretreatment (Fig. 2, S2-1, S2-3, S2-5, and S2-6). The maximum differences in the cell death rate caused by the IAA pretreatment and the control (cell death rate with the IAA pretreatment minus that in the control), which ranged between –0.755 and –0.122, were detected after 9 h in most of the examined strains (S288c, UWOPS83-787.3, and Y12), but at 15 h in Sigma 1278b. In contrast, two strains (378604X and YS9) showed no significant or slightly greater (cell death rate with the IAA pretreatment minus that in the control=0.097) changes in the cell death rate under the same treatment (Fig. 2, S2-2, and S2-4).

### IAA-induced ethanol tolerance was detected in some Saccharomycetales yeast strains based on markedly higher colony numbers on plates

**Fig. 3.**
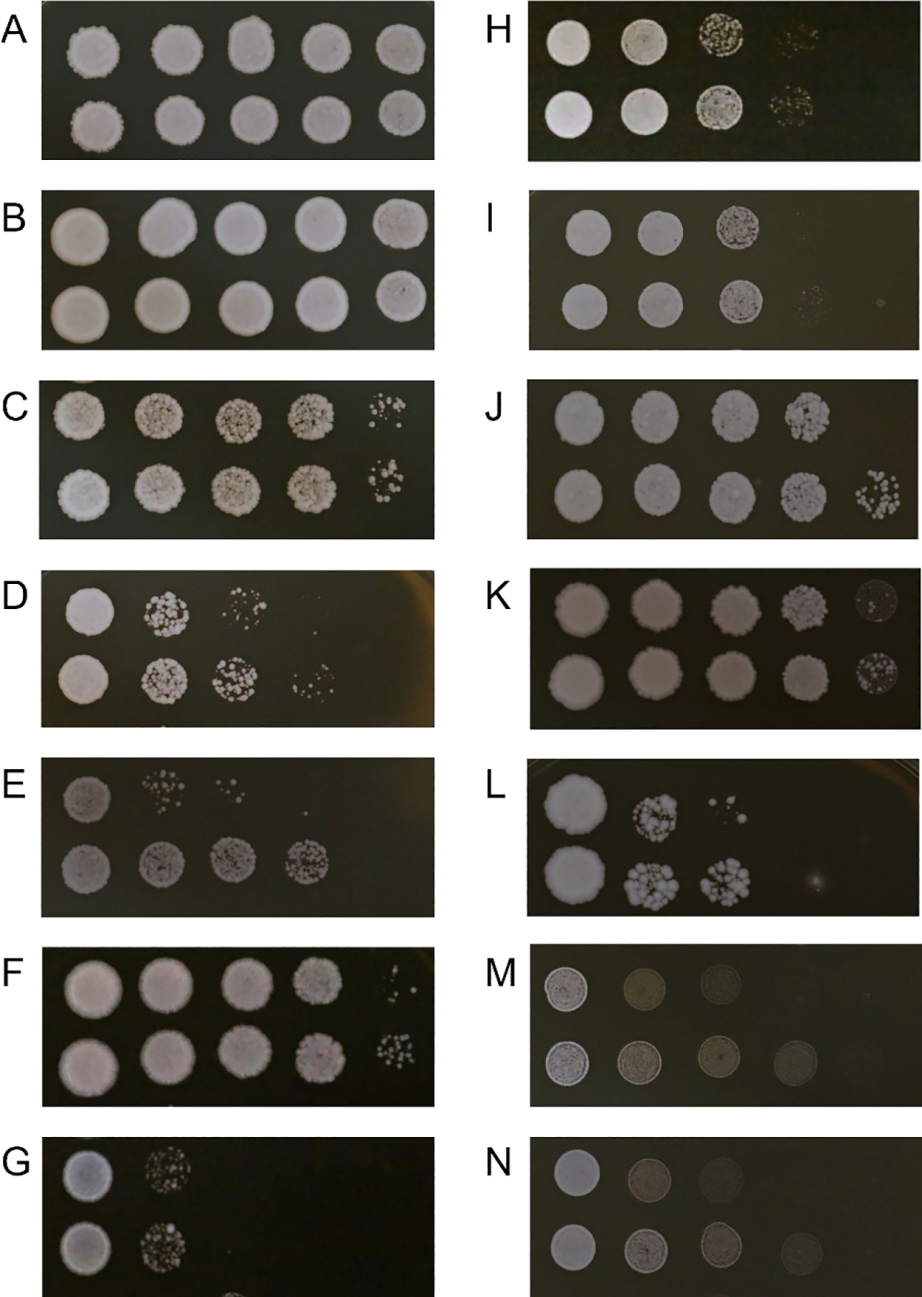
Saccharomycetales yeast growth on yeast extract–peptone–dextrose (YPD) agar plates to check the effects of the indole-3-acetic acid (IAA) pretreatment on the cell density of yeasts cultured with YPD media containing 13% ethanol. In each image, yeast was pretreated without IAA (top row) or with IAA (bottom row) and spotted onto YPD agar plates after culturing with ethanol for 0, 4, 8, 12, and 24 h (from left to right). The presence of growth at later time points indicates increased ethanol tolerance. A: *Saccharomyces cerevisiae* UWOPS83-2421; B: *S. paradoxus* N-17; C: *S. paradoxus* CBS5829; D: *S. eubayanus* Sgn 25; E: *S. eubayanus* YDG186; F: *Kazachstania servazzii* JYC2565; G: *K. servazzii* JYC2573; H: *Zygosaccharomyces bisporus* JYC 2526; I: *Z. rouxii* JYC 2561; J: *Torulaspora* sp. JYC 369; K: *Kluyveromyces marxianus* JYC2528; L: *Kluyveromyces* sp. JYC527; M: *Dekkera bruxellensis* JYC2592; N: *D. bruxellensis* JYC2595.

IAA-induced ethanol tolerance was detected in some Saccharomycetales yeast strains based on markedly higher colonies growing on plates. In comparisons with the control, a higher number of cell colonies was found in IAA-pretreated Saccharomycetales yeasts in at least 4 strains that were cultured in media containing 11% ethanol (*S. paradoxus* CBS5829; *Z. rouxii* JYC 2561; *Torulaspora* sp. JYC 369; and *Kluyveromyces* sp. JYC527) (Fig. S3), and at least 11 strains that were cultured in media containing 13% ethanol (*S. paradoxus* CBS5829; *S. eubayanus* Sgn 25; *S. eubayanus* YDG186; *Ka. servazzii* JYC2565; *Z. bisporus* JYC 2526; *Z. rouxii* JYC 2561; *Torulaspora* sp. JYC 369; *Kl. marxianus* JYC2528; *Kluyveromyces* sp. JYC527; *D. bruxellensis* JYC2592; and *D. bruxellensis* JYC2595) (Fig. 3). Therefore, IAA-induced ethanol tolerance was detected in different Saccharomycetales yeast genera, but varied with the concentration of ethanol.

## Discussion

IAA has been suggested to function as a widespread physiological code in interactions between fungi and other organisms. Fungal-produced IAA has been suggested to play roles in signaling pathogenic or symbiotic plant interactions (Spaepen and Vanderleyden, 2011). In fungal-fungal interactions, IAA has also been proposed to function as a quorum-sensing signal that regulates virulence traits, such as hyphal transition, in many pathogenic fungi. It also influences competition between fungal species that occupy the same niche (Fu *et al.*, 2015). However, limited information is currently available on the roles of IAA in fungal-microbial interactions. In the present study, exogenous IAA exerted significant effects on some *S. cerevisiae* yeast strains responding to environmental stresses. The IAA pretreatment promoted cell growth in some *S. cerevisiae* strains exposed to certain stress conditions, similar to that reported in the bacteria *Rhizobium* and *E. coli* (Bianco *et al.*, 2006; Donati *et al.*, 2013). However, IAA failed to induce significant effects in most of the yeast strains grown under heat or osmotic stress. The effects of IAA may be dependent on the dose and strain (Sun *et al.*, 2014). The concentration of IAA naturally produced by fungi generally ranges between approximately 60 and less than 1,400‍ ‍μM (Sun *et al.*, 2014; Liu *et al.*, 2016). The addition of exogenous IAA may reduce the yeast growth rate (Prusty *et al.*, 2004; Liu *et al.*, 2016) or promote it at relatively low concentrations (Kulkarni *et al.*, 2013; Sun *et al.*, 2014). The duration of the IAA pretreatment may also be a factor. The durations of IAA pretreatments used in previous studies varied by organism, with durations of 2 h for prokaryotic bacteria (Bianco *et al.*, 2006), 3 h for rhizobia (Donati *et al.*, 2013), 24 h for algae (Lin *et al.*, 2020), and 12 h for yeast (Sun *et al.*, 2014). The durations of the IAA pretreatment used in the present study (24 and 48 h) were sufficient to trigger ethanol tolerance; however, further studies are needed to clarify how the duration of the pretreatment may affect this response.

In the present study, the IAA pretreatment exerted the greatest effects on *S. cerevisiae* cultures grown under ethanol stress. Ethanol affects numerous cellular behaviors and processes related to cell death, including necrosis and apoptosis (Kitagaki *et al.*, 2007). Increases in ethanol density gradually reduce cell viability by influencing the integrity and function of the cell membrane (Ding *et al.*, 2009). *Saccharomyces* yeasts produce ethanol following the degradation of sugars under aerobic conditions, which is known as the Crabtree effect, to outcompete bacteria (Rozpędowska *et al.*, 2011). However, ethanol negatively affects *S. cerevisiae *itself (Auesukaree, 2017; Morard *et al.*, 2019). Under stress conditions, microorganisms engage in a number of behaviors to prevent denaturation and stabilize cell membranes, including the secretion of extracellular polysaccharides, formation of biofilms, accumulation of trehalose, or aggregation of membrane proteins (Pereira and Hunenberger, 2008). In *S. cerevisiae*, ethanol tolerance is achieved by the intracellular accumulation of trehalose (Sharma, 1997) or the production of unsaturated fatty acids and ergosterol to increase cell membrane stability (Daum *et al.*, 1998; Swan and Watson, 1998; You *et al.*, 2003). In the present study, we found the IAA pretreatment reduced the lethal effects of ethanol on yeast cells. One possible reason is that the IAA pretreatment triggered self-protection mechanisms for *S. cerevisiae* growth in the presence of ethanol. A previous study demonstrated that IAA regulated cell death–related genes by inducing the expression of heat-shock proteins, cold-shock proteins, and molecular chaperones, all of which help microorganisms overcome stress (Donati *et al.*, 2013). Another possible reason for the lower cell death rate is that IAA enhances the metabolism of ethanol. The make-accumulate-consume strategy provides *S. cerevisiae* with the capability to metabolize ethanol. Enhanced metabolism may reduce ethanol concentrations and rescue cells from death. Although we herein did not measure the final concentration of ethanol in culture media, we cannot exclude the possibility that a decrease in ethanol was one of the reasons for a lower cell death rate.

In the present study, we detected IAA-induced ethanol tolerance in Saccharomycetales yeasts. However, IAA is not a byproduct of ethanol production, it is more likely to be an indicator of sympatric yeast competitors associated with the accumulation of ethanol. The ecological and evolutionary significance of IAA-induced ethanol tolerance remains unclear. Ethanol is toxic to Saccharomycetales yeasts, but is also a strategy for outcompeting sympatric microbes (Piškur *et al.*, 2006). Therefore, although further studies are warranted, we herein hypothesize that IAA-induced ethanol tolerance evolved under the close association of IAA produced by sympatric microbes and the consequent accumulation of ethanol. There is fierce competition among microbes for the high-energy-density carbon resources provided by sugar-rich fruits. In Saccharomycetales yeasts, the ability to produce ethanol evolved as a means to outcompete other microorganisms and predominate high-sugar niches (Piškur *et al.*, 2006). Ethanol production has been reinforced in *S. cerevisiae* and its close relatives after genome duplication events that hypothetically occurred at the same time as the appearance of angiosperms (Dashko *et al.*, 2014). In Saccharomycetales yeasts, an elevated IAA concentration may reflect increasing numbers of competitive microorganisms, which are consequently associated with the accumulation of ethanol. In the present study, we found that IAA protected against ethanol-related cell death in at least six genera (*Dekkera*, *Kazachstania*, *Kluyveromyces*, *Saccharomyces*, *Torulaspora*, and *Zygosaccharomyces*) belonging to the families Pichiaceae and Saccharomycetaceae. These results are consistent with the hypothesis that ethanol production is a commonly evolved strategy in Saccharomycetales yeasts (Dashko *et al.*, 2014). The make-accumulate-consume strategy has evolved convergently in *S. cerevisiae* and *Dekkera* spp., and endows these Saccharomycetales yeasts with greater ethanol tolerance (Rozpędowska *et al.*, 2011). Ethanol accumulation is a main stressor during fermentation in sugar-rich niches. Although *S. cerevisiae* and *Dekkera* are more tolerant of ethanol than are other Saccharomycetales yeasts, pre-WGD and post-WGD Saccharomycetales yeasts both generally displayed higher ethanol tolerance following IAA pre-exposure. Further studies are needed to elucidate how IAA is associated with specific alcohol stress–signaling mechanisms in these yeasts.

In conclusion, we herein characterized the effects of IAA on stress tolerance in several Saccharomycetales yeasts. Among the three stress conditions examined, ethanol stress was the most prominently affected by IAA inducing ethanol tolerance, as shown by the attenuation of cell death in the presence of ethanol. Ethanol production helps Saccharomycetales yeasts to outcompete sympatric bacteria that occupy sugar-rich niches. The presence of IAA, which accompanies an increased density of microorganisms, may reflect intense competition and associated ethanol accumulation. Therefore, IAA-induced self-protection occurs not only in *S. cerevisiae*, in which the wine trait has been reinforced during evolution, but also generally in Saccharomycetales yeasts that are both phylogenetically related and distinct. This stress tolerance mechanism reveals that Saccharomycetales yeasts have an inherent ability to increase their stress response once appropriate external and/or internal triggers are activated. Some genes have been implicated in ethanol tolerance by examinations of hybrid strains (*e.g*., Lancaster *et al.*, 2019; Lairón-Peris *et al.*, 2020). As one of a few environmental factors that trigger ethanol tolerance in Saccharomycetales yeasts, the built‐in molecular mechanisms underlying stress responses warrant further study.

## Citation

Hsiung, R.-T., Chiu, M.-C., and Chou, J.-Y. (2022) Exogenous Indole-3-Acetic Acid Induced Ethanol Tolerance in Phylogenetically Diverse Saccharomycetales Yeasts. *Microbes Environ ***37**: ME21053.

https://doi.org/10.1264/jsme2.ME21053

## Supplementary Material

Supplementary Material

## Figures and Tables

**Table 1.** Significance of effects of the IAA pretreatment

Stress	Yeast strain	Significance of the IAA pretreatment^1^				Significance of interactions in the effects of stress on the IAA pretreatment^2^		
		*d.f.*	*F*	*P*		*d.f.*	*F*	*P_int*
No stress	273614N	4	1.382	0.270		4	**—**	**—**
	322134S	4	1.171	0.348		4	**—**	**—**
	378604X	4	0.386	0.817		4	**—**	**—**
	DBVPG1106	4	0.285	0.885		4	**—**	**—**
	NCYC110	4	1.770	0.168		4	**—**	**—**
	S288c	4	0.525	0.718		4	**—**	**—**
	Sigma 1278b	4	3.297	**0.027** ^3^		4	**—**	**—**
	UWOPS83-787.3	4	0.214	0.928		4	**—**	**—**
	UWOPS87-2421	4	0.282	0.887		4	**—**	**—**
	Y12	4	2.913	**0.043** ^3^		4	**—**	**—**
	Yllc17_E5	4	0.170	0.952		4	**—**	**—**
	YS9	4	4.207	**0.010** ^3^		4	**—**	**—**
Ethanol	273614N	4	0.831	0.519		4	0.575	0.682
	322134S	4	2.003	0.126		4	1.386	0.253
	378604X	4	5.243	**0.004** ^3^		4	4.016	**0.006** ^3^
	DBVPG1106	4	1.734	0.175		4	0.968	0.434
	NCYC110	4	0.614	0.656		4	0.280	0.889
	S288c	4	3.069	**0.036** ^3^		4	2.574	**0.049** ^3^
	Sigma 1278b	4	7.947	**<0.001** ^3^		4	5.502	**<0.001** ^3^
	UWOPS83-787.3	4	4.70	**0.006** ^3^		4	2.934	**0.030** ^3^
	UWOPS87-2421	4	0.969	0.443		4	0.929	0.455
	Y12	4	3.157	**0.031** ^3^		4	2.942	**0.029** ^3^
	Yllc17_E5	4	1.834	0.155		4	1.584	0.193
	YS9		3.280	**0.028** ^3^		4	0.710	0.589
Heat	273614N	4	3.375	**0.025** ^3^		4	2.408	0.062
	322134S	4	1.727	0.177		4	2.063	0.100
	378604X	4	0.581	0.680		4	0.398	0.809
	DBVPG1106	4	0.596	0.669		4	0.488	0.745
	NCYC110	4	0.830	0.519		4	0.345	0.846
	S288c	4	1.258	0.313		4	0.928	0.455
	Sigma 1278b	4	1.161	0.351		4	1.343	0.267
	UWOPS83-787.3	4	2.093	0.113		4	1.850	0.134
	UWOPS87-2421	4	1.020	0.417		4	0.889	0.478
	Y12	4	1.328	0.288		4	0.726	0.579
	Yllc17_E5	4	1.423	0.255		4	0.970	0.432
	YS9	4	4.212	**0.010** ^3^		4	3.381	**0.016** ^3^
Osmotic	273614N	4	0.701	0.599		4	0.362	0.835
	322134S	4	1.315	0.293		4	1.232	0.310
	378604X	4	0.008	0.999		4	0.009	0.999
	DBVPG1106	4	0.343	0.846		4	0.215	0.929
	NCYC110	4	0.338	0.849		4	0.310	0.870
	S288c	4	1.357	0.278		4	1.179	0.331
	Sigma 1278b	4	2.773	**0.049** ^3^		4	2.572	**0.049** ^3^
	UWOPS83-787.3	4	1.277	0.307		4	0.812	0.524
	UWOPS87-2421	4	1.447	0.249		4	0.979	0.428
	Y12	4	0.471	0.757		4	0.595	0.668
	Yllc17_E5	4	3.272	**0.028** ^3^		4	1.523	0.210
	YS9	4	1.042	0.406		4	0.540	0.707

^1^ Significance of the fixed effect in the one-factor LMM model with IAA titers as the fixed factor variable. ^2^ Significance of the interaction term of the fixed factor variables, IAA titers and environmental stress, in the two-factor LMM model. ^3^ Significance of the IAA pretreatment and the interaction between the IAA pretreatment and environmental stress, as indicated by **bold** *P* values.
