# Supplementary material for: Exogenous Indole-3-Acetic Acid Induced Ethanol Tolerance in Phylogenetically Diverse Saccharomycetales Yeasts
Source: Microbes Environ. 2022 Jan 27;37(1):ME21053. doi: 10.1264/jsme2.ME21053 (PMC8958292; doi:10.1264/jsme2.ME21053)
Supplement: Supplementary file 1 — Supplementary Material [file 37_21053_s1.pdf]

Supplementary Table 1. *Saccharomyces cerevisiae* strains and other yeast used in this study.

Strains	Source
Saccharomyces cerevisiae [1]	
S288c	Rotting fig
Sigma 1278b	Undescribed in Grenson *et al.* (1966)[6]
322134S	Clinical isolate (throat sputum)
378604X	Clinical isolate (sputum)
273614N	Clinical isolate (fecal)
YS9	Baker strain
UWOPS83-787.3	Fruit, *Opuntia stricta*
UWOPS87-2421	Cladode, *Opuntia megacantha*
DBVPG1106	Grapes
Yllc17_E5	Wine
Y12	Palm wine strain
NCYC110	Ginger beer from *Z. officinale*
Saccharomyces paradoxus [1]	
N-17	Exudate of *Quercus robur*
CBS5829	Mor soil, pH 3.6
Saccharomyces eubayanus [2]	
Sgn 25	Undescribed in Greig *et al.* (2002)[2]
YDG186	CBS, Utrecht, The Netherland
Kazachstania servazzii [3]	
JYC2565	Milk kefir
JYC2573	Milk kefir
Zygosaccharomyces bisporus [4]	
JYC 2526	Coffee cherry
Zygosaccharomyces rouxii [3]	
JYC 2561	Fermented vinegar
***Torulaspora* sp.** [5]	
JYC 369	*Drosera spatulata*
Kluyveromyces marxianus [3]	
JYC2528	Milk kefir
***Kluyveromyces* sp.** [4]	
JYC527	Rhizosphere of cherry tomato (*Lycopersicon esculentum* var.

	cerasiforme)
Dekkera bruxellensis [4]	
JYC2592	Kombucha culture
JYC2595	Kombucha culture

[1] Liti, G., Carter, D. M., Moses, A. M., Warringer, J., Parts, L., James, S. A., et al. (2009) Population genomics of domestic and wild yeasts. *Nature* 458, 337-341. doi: 10.1038/nature07743

[2] Greig, D., Borts, R. H., Louis, E. J., and Travisano, M. (2002). Epistasis and hybrid sterility in *Saccharomyces*. *Proc. Royal Soc. B* 269, 1167-1171. doi: 10.1098/rspb.2002.1989

[3] Hsu, S. A., and Chou, J. Y. (2021). Yeasts in fermented food and kefir: In vitro characterization of probiotic traits. *J. Anim. Plant Sci.* 31, 567-582. doi: 10.1016/j.lwt.2015.07.042

[4] Unpublished data

[5] Fu, S. F., Sun, P. F., Lu, H. Y., Wei, J. Y., Xiao, H. S., Fang, W. T. et al. (2016). Plant growth-promoting traits of yeasts isolated from the phyllosphere and rhizosphere of *Drosera spatulata* Lab. *Fungal Biol.* 120, 433-448. doi: 10.1016/j.funbio.2015.12.006

[6]Grenson, M., Mousset, M., Wiame, J. M., and Bechet, J. (1966). Multiplicity of the amino acid permeases in *Saccharomyces cerevisiae*. Biochimica *et* Biophysica Acta (BBA)-General Subjects 127: 325-338. doi:10.1016/0304-4165(66)90387-4

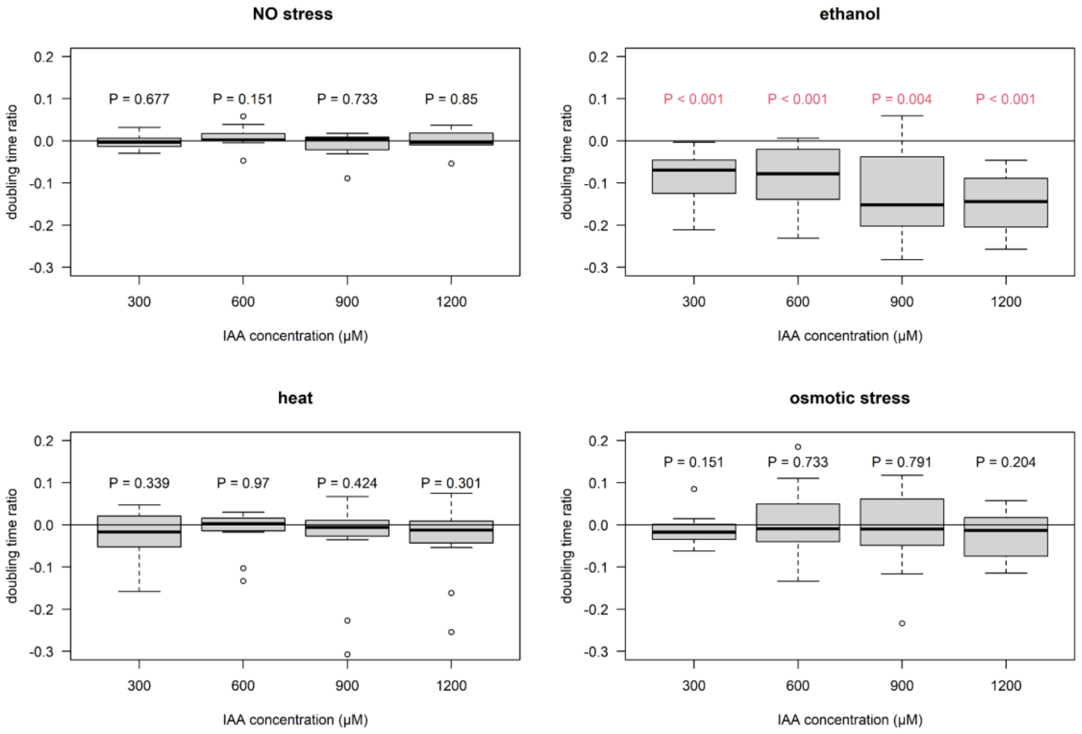

Supplementary Figure 1. Doubling time ratio of 12 *Saccharomyces cerevisiae* strains in response to three examined stresses (ethanol, heat, and osmotic) after pretreatment with various indole-3-acetic acid (IAA) titers. Values of the doubling time ratio fall within the range of -1 to 1, where 0 represents no effect on stress tolerance. *P* values were calculated using a Mann–Whitney *U* test to indicate the significance of the doubling time ratio compared to 0 (black line).

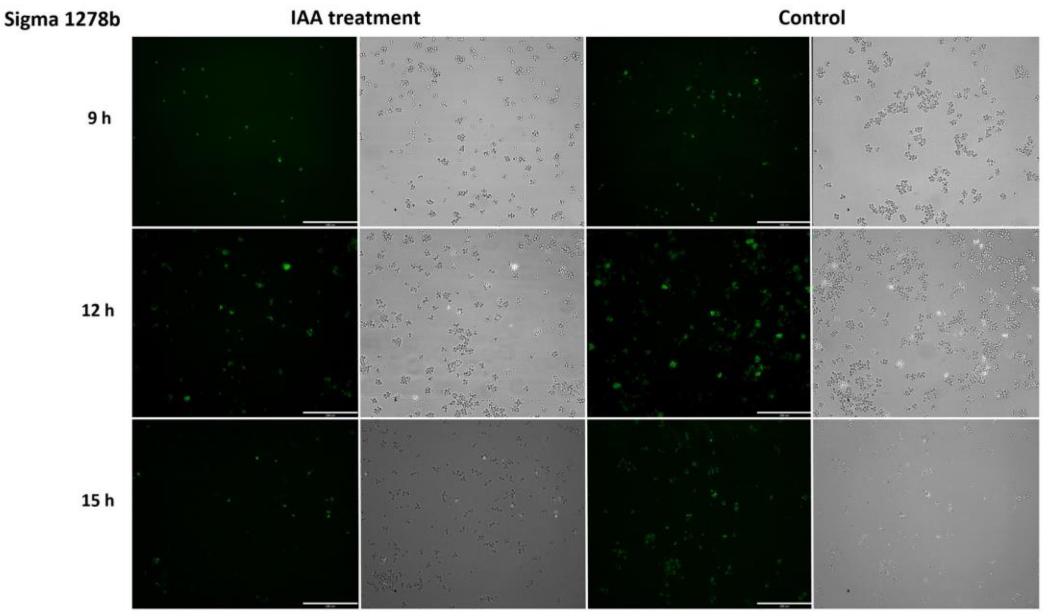

Supplementary Figure 2-1. Fluorescence image of the *Saccharomyces cerevisiae* strain Sigma 1278b cultured in ethanol as an environmental stress. IAA treatment: indole-3-acetic acid at 900 uM and ethanol at 11 %; control treatment: ethanol only at 11 %.

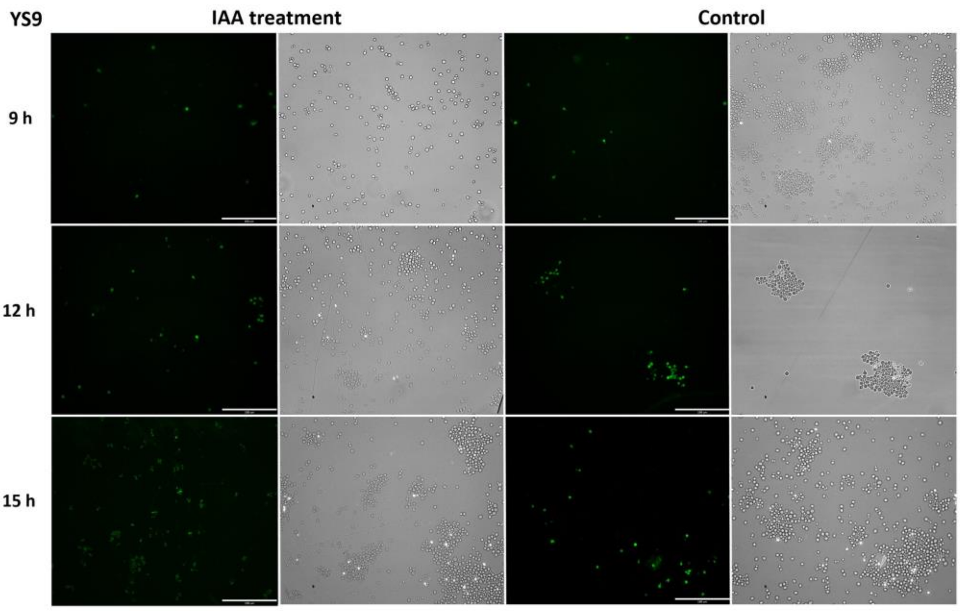

Supplementary Figure 2-2. Fluorescence image of the *Saccharomyces cerevisiae* strain YS9 cultured in ethanol as an environmental stress. IAA treatment: indole-3-acetic acid at 900 uM and ethanol at 11 %; control treatment: ethanol only at 11 %.

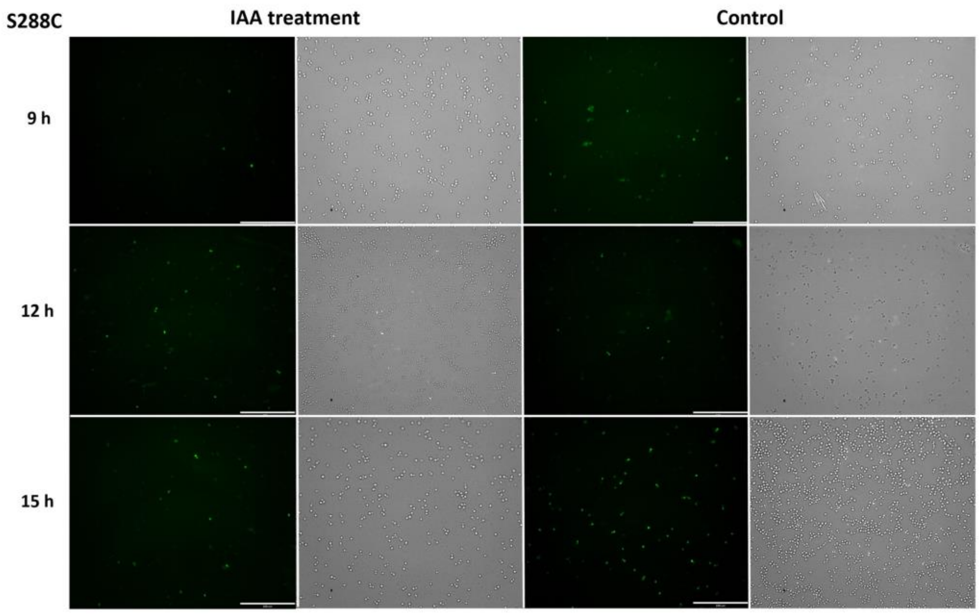

Supplementary Figure 2-3. Fluorescence image of the *Saccharomyces cerevisiae* strain S288c cultured in ethanol as an environmental stress. IAA treatment: indole-3-acetic acid at 900 uM and ethanol at 11 %; control treatment: ethanol only at 11 %.

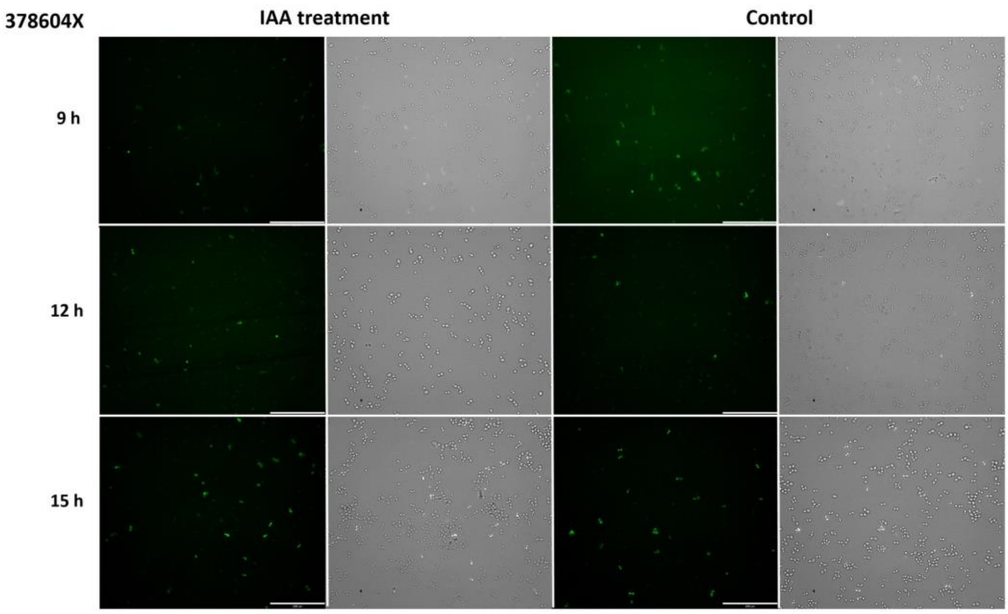

Supplementary Figure 2-4. Fluorescence image of the *Saccharomyces cerevisiae* strain 378604x cultured in ethanol as an environmental stress. IAA treatment: indole-3-acetic acid at 900 uM and ethanol at 11 %; control treatment: ethanol only at 11 %.

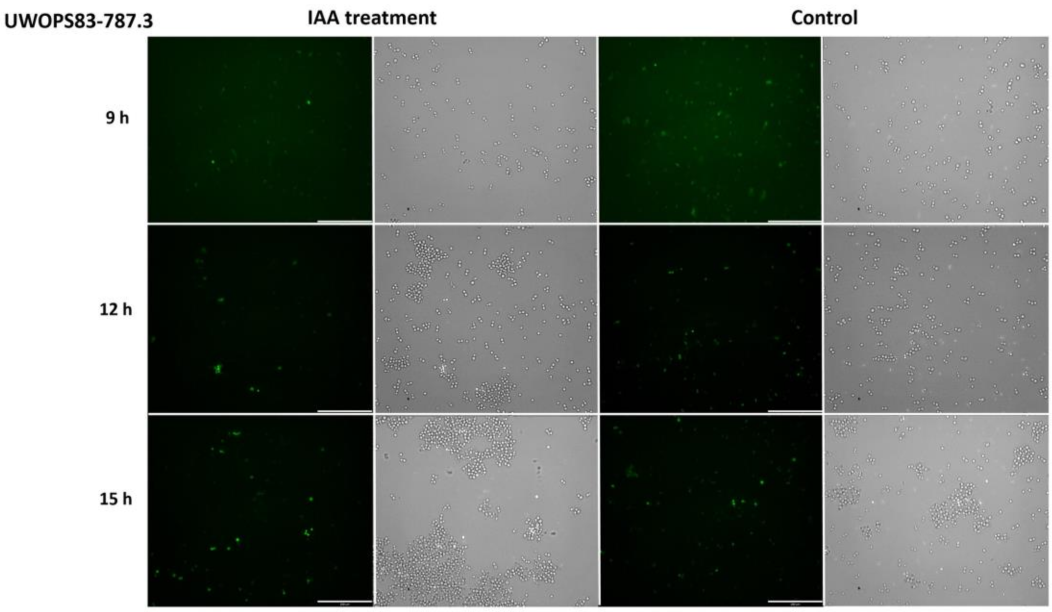

Supplementary Figure 2-5. Fluorescence image of the *Saccharomyces cerevisiae* strain UWOPS83-787.3 cultured in ethanol as an environmental stress. IAA treatment: indole-3-acetic acid at 900 uM and ethanol at 11 %; control treatment: ethanol only at 11 %.

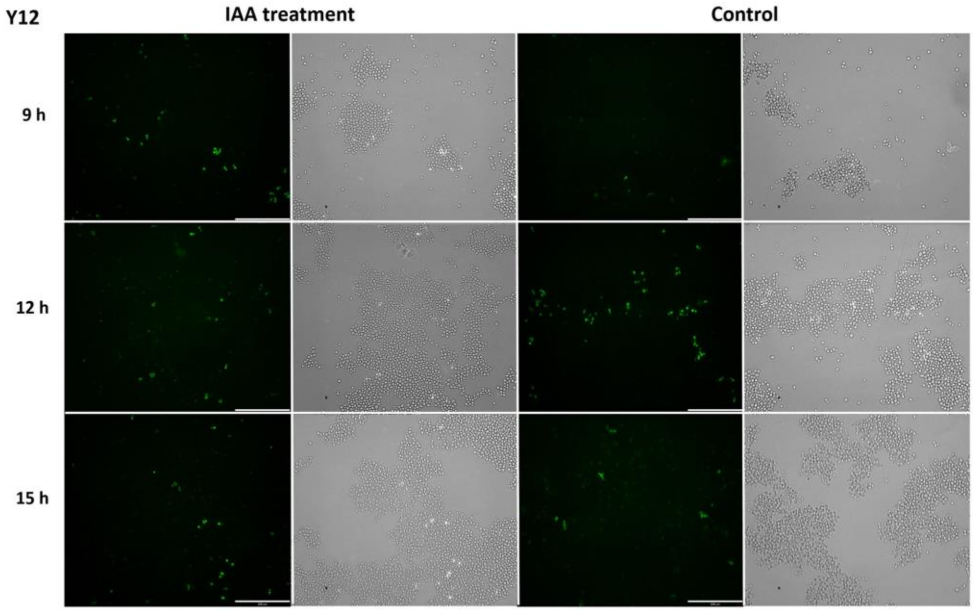

Supplementary Figure 2-6. Fluorescence image of the *Saccharomyces cerevisiae* strain Y12 cultured in ethanol as an environmental stress. IAA treatment: indole-3-acetic acid at 900 uM and ethanol at 11 %; control treatment: ethanol only at 11 %.

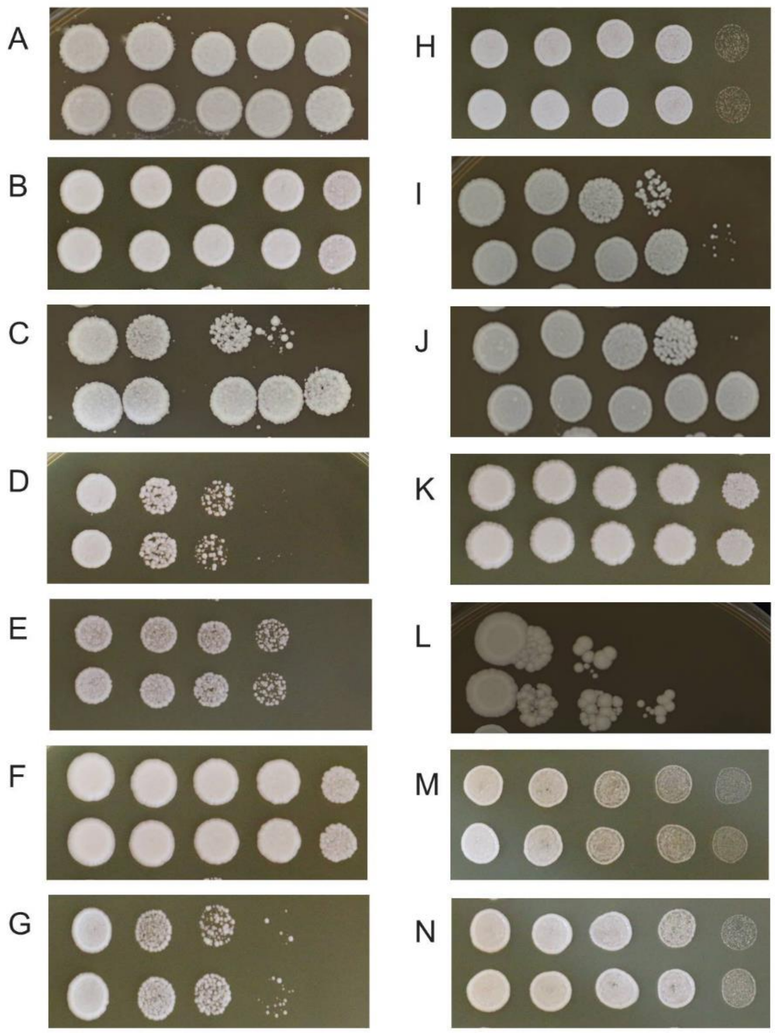

Supplementary Figure 3. Saccharomycetales yeast growth on yeast extract–peptone–dextrose (YPD) agar plates to check the cell density of the effect of indole-3-acetic acid (IAA) pretreatment on the yeasts cultured with YPD media containing 11% ethanol. In each image, yeast was pretreated without IAA (top row) or with IAA (bottom row) and spotted onto YPD agar plates after culturing with ethanol for 0, 4, 8, 12, and 24 h (from left to right). The presence of growth at later time points indicates increased ethanol tolerance. A: *Saccharomyces cerevisiae* UWOPS83-2421; B: *S. paradoxus* N-17; C: *S. paradoxus* CBS5829; D: *S. eubayanus* Sgn 25; E: *S. eubayanus* YDG186; F: *Kazachstania servazzii* JYC2565; G: *K. servazzii* JYC2573; H: *Zygosaccharomyces bisporus* JYC 2526; I: *Z. rouxii* JYC 2561; J: *Torulaspora* sp. JYC 369; K: *Kluyveromyces marxianus* JYC2528; L: *Kluyveromyces* sp. JYC527; M: *Dekkera bruxellensis* JYC2592; N: *D. bruxellensis* JYC2595.
